# Risk Assessment of Metals in Urban Soils from a Typical Industrial City, Suzhou, Eastern China

**DOI:** 10.3390/ijerph14091025

**Published:** 2017-09-07

**Authors:** Gang Wang, Hou-Qi Liu, Yu Gong, Yang Wei, Ai-Jun Miao, Liu-Yan Yang, Huan Zhong

**Affiliations:** 1 State Key Laboratory of Pollution Control and Resource Reuse, School of the Environment, Nanjing University, Nanjing 210023, Jiangsu, China; gwang@189.cn (G.W.); gongyu111@hotmail.com (Y.G.); miaoaj@nju.edu.cn (A.-J.M.); 2 Suzhou Institute of USTC, Suzhou 215123, Jiangsu, China; lhq-1981@163.com; 3 Institute for Advanced Study, Shenzhen University, Shenzhen 518060, China; weiyang@szu.edu.cn

**Keywords:** metals, urban soil, ecological risk, health risk

## Abstract

Risk of metals in urban soils is less studied, compared to that in other types of soils, hindering accurate assessment of human exposure to metals. In this study, the concentrations of five metals (As, Cd, Cr, Pb, and Hg) were analyzed in 167 surface soil samples collected from Suzhou city and their potential ecological and human health risks were assessed. The mean concentrations of As, Cd, Pb, and Hg except Cr, were higher than the background values in Jiangsu Province. Metal concentrations varied among districts, where sites of high contamination showed a punctate distribution. Principal components and correlation analyses revealed that As, Pb, and Cd could originate from the same sources. The geo-accumulation (I_geo_) and potential ecological risk indices (RI) were calculated and the relatively low values of I_geo_ (<0) and RI (<150) suggested generally low ecological risk. The noncarcinogenic risks of the metals were relatively low for Suzhou residents (i.e., average hazard index or HI: 0.1199 for adults and 0.5935 for children, <1), while the total carcinogenic risks (TCR) of Cr and As were acceptable (TCR in the range of 1.0 × 10^−6^ to 1.0 × 10^−4^). Children faced a higher threat than adults. Results of Monte-Carlo simulations were lower than those obtained from models using deterministic parameters. Of all the uncertain parameters, the ingestion rate and body weight were the most sensitive for adults and children, respectively, while As was an important factor for both. The results as well as the factors controlling risks of metals could help better understand the risks of metals in urban soils of industrial cities in China.

## 1. Introduction

Pollution of soil by metals in China and the associated ecological and health risks are currently matters of increasing concern. According to a recent national survey conducted in 2014 by the Ministry of Environmental Protection and the Ministry of Land and Resources of China [1], 7.0%, 1.6%, 2.7%, 2.1%, 1.5%, 1.1%, 0.9%, and 4.8% of soils in China exceed the limit for Cd, Hg, As, Cu, Pb, Cr, Zn, and Ni, respectively. Recently, considerable progress has been made in understanding the risks posed by metals in soils in China, as evidenced by the rapid increases in the number of annual scientific publications addressing this topic (Figure S1). While much attention has been paid to the risk of metals in agricultural soils and industrial land, less is known about metal pollution in Chinese urban soils (Figure S1). This information is critical, considering the continuous exposure of urban residents, and especially children, to the metals in urban soils, whether through oral ingestion, inhalation, or dermal contact [2,3,4,5,6]. As in agricultural and industrial soils, urban soils are subject to metal inputs from atmospheric deposition, irrigation water, industrial activities, and natural sources [7,8,9,10,11]. Meanwhile, the pollution of urban soils with Pb, As, and Cr could also derive from vehicle exhaust and municipal solid waste [12,13]. 

The few published studies on the pollution of urban soils in China have reported elevated concentrations of metals [14,15,16,17,18,19,20] as well as the associated high ecological and/or health risks. For example, a significant potential health risk associated with soils was determined in an industrial district in Tianjin [21]. The risk index (RI) values of topsoil samples in a county in Shanxi Province were >600 and the total carcinogenic risks (TCR) due to As, Cr, and Ni for children were higher than the threshold value (1.0 × 10^−4^) [22]. However, while those previous studies largely focused on a single type of urban soil, comprehensive risk assessments of metals in the major types of urban soils in China are still lacking, as far as we know.

Roadside, residential, and park soils are the most common types of urban soils that expose local residents to metals. Metal (especially Cu, Zn, Cd, and Pb) contamination in roadside soils has received the most attention [23,24,25,26,27,28,29] because of the concerns about metal release from fuel combustion [30,31]. By contrast, the risks posed by metals in residential and park soils are less understood. Residential soils are subject to metal inputs from wood preservatives and vehicle emissions, among other sources [32]. Park soils can become contaminated either directly or by metals bound to airborne particles released by vehicle and industrial sources in adjacent areas [33,34]. For example, soils in a residential zone and park built on a brownfield left by a smelting plant were shown to contain high levels of metals [35]. Similar investigations of metal contamination in residential and park soils have been conducted in the USA [32,36], Spain [37], and Serbia [38]. However, in China, little is known about metal contamination in residential and park soils nor about the risks related to these types of urban soils.

The main objective of this study is to assess the risks posed by metal contamination of different types of urban soils from Suzhou, a typical industrial city in eastern China. Among cities of the Yangtze River delta (the most developed area in China), Suzhou has the second largest population and the second highest GDP. As such, it is representative of cities impacted by industrial processes in China. Surface soil samples were collected from roadside, residential, and park sites in Suzhou city and analyzed for their metal concentrations. The ecological risk of metals in soils was assessed by calculating the geo-accumulation index [39,40] and the potential ecological risk index [41]. The associated health risk was then assessed by determining metal exposure through oral ingestion, inhalation, and dermal contact and calculating the hazard quotient (HQ), hazard index (HI), and carcinogenic risk (CR). The results of our study will contribute to a more comprehensive understanding of the risks arising from human exposure to metals in Chinese cities.

## 2. Materials and Methods 

### 2.1. Sampling and Metal Analysis

**Figure 1:**
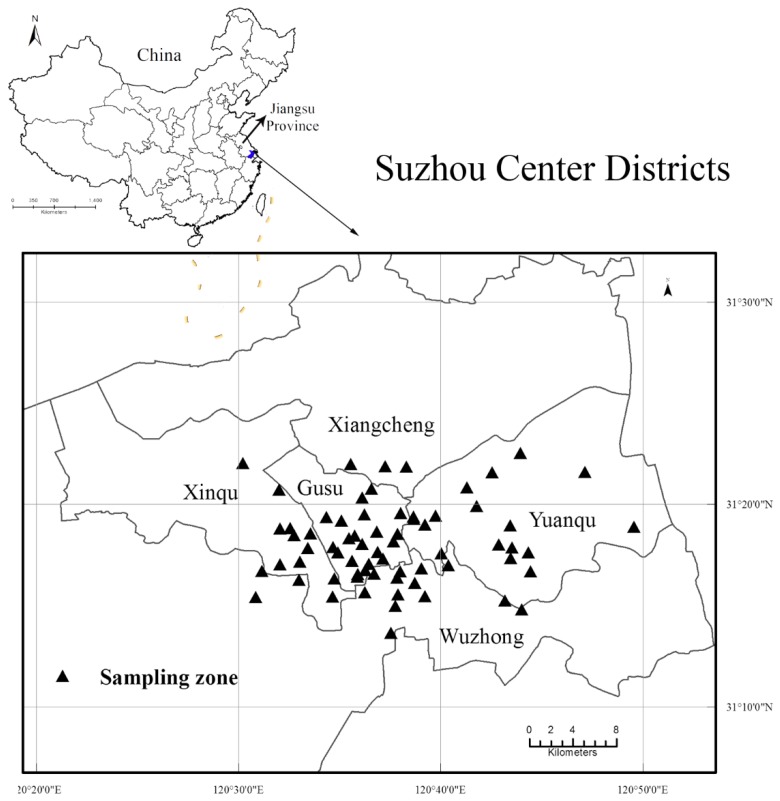
Sampling zones in Suzhou center districts, Jiangsu, China.

The study was performed in Suzhou, an industrial city and one of the major cities in Jiangsu Province, in November 2016. Sixty eight sampling zones were identified in different districts of Suzhou. Three major types of urban soils were considered, including soil by roadside, soil in residential quarter, and soils in the park with lots of trees. Among the sampling zones, 31 zones included all three types of soils, and the rest with roadside and residential soils only. In each sampling zone, 2−3 sampling sites were selected, with each site for a specific type of soil, i.e., roadside, residential and park. All soil samples were collected from the same layer, i.e., surface soil at 5−15 cm. Thus, depth-dependent categorization of samples were not necessary in this study, also considering the generally reported less-variable or uniform distribution of metals in surface soils in urban areas [42,43,44]. At each site, three surface soil subsamples (100−200 g) were collected using stainless steel spade along the diagonal and pooled into one sample [45]. Thus a total of 167 soil samples, including 68 samples from roadside, 68 samples from residential and 31 samples from park, were obtained (Figure 1).

After removing gravel and grasses, the soil samples were air-dried, ground, sieved through an 0.2 mm-mesh and digested with HNO_3_:HClO_4_:HF (3:1:1, v/v), (Sinopharm, Shanghai, China) and the concentrations of metals were then determined using inductively coupled plasma atomic emission spectrometry (PerkinElmer Optima 8300, Waltham, MA, USA). The analytical quality controls include blanks, duplicate samples, and certified reference material (i.e., reference soil, Beijing, China, GBW07401). The recoveries of metals in the certified reference material ranged from 88% to 110%.

### 2.2. Ecological Risk Assessment

#### 2.2.1. Geo-Accumulation Index (I_geo_)

The geo-accumulation index (I_geo_) was used to assess contamination of a specific metal in soils by evaluating metal enrichment above baseline or background values. The geo-accumulation index was calculated according to Equation (1) [46,47]:

(1) $$I_{\mathrm{geo}}=\log_{2}\left({\mathrm{EF}}_{n}/1.5\right)=\log_{2}\left(C_{n}/1.5\times B_{n}\right)$$

where EF_n_ is the enrichment factor for given metal, C_n_ is the measured metal concentration (mg/kg), B_n_ is the background value of the metal concentrations (mg/kg) in soils of Jiangsu Province [48], i.e., As, 10 mg/kg, Pb 26.2 mg/kg, Hg 0.29 mg/kg, Cr 77.8 mg/kg, and Cd 0.13 mg/kg. For the geo-accumulation index, metal pollution levels are classified as: Table 1. 

#### 2.2.2. Potential Ecological Risk Index

The potential ecological RI, a common method of ecological risk assessment [49,50], was adopted to evaluate the overall ecological risks of metal pollution in Suzhou soils. This quantitative approach, developed by Hakanson [41], includes the toxic response factor (T_r_) so that the toxicity of each metal is taken into account. The potential ecological risk factor of a given metal (E_r_) was introduced before calculating the RI of all the metals in the soils.

(2) $$E_{r}=T_{r}\times C_{f}=T_{r}\times C_{i}/C_{0}$$

(3) $$\mathrm{RI}=\overset{n}{\underset{i=1}{\sum }}E_{r}$$

where C_f_ corresponds to the toxic response of a specific metal, which is obtained by dividing the metal concentration by its background level [48]. The T_r_ of the studied metals was 2 for Cr, 5 for Pb, 10 for As, 30 for Cd, and 40 for Hg [41]. The ecological risk of each metal corresponds to its E_r_ value and the RI value indicates the ecological risk of all metals in the soil (Table 1).

### 2.3. Health Risk Assessment

#### 2.3.1. Exposure Assessment

For metals in soil, there are three pathways by which humans may be exposed: ingestion, inhalation, and dermal contact [51]. These indicators are commonly used in assessing risk of metals in both urban and rural areas of China **[22,52,53,54],** especially, ingestion of soil particles have been found to be an unneglectable pathway of human exposure to metals for urban populations [55,56]. The average daily intake (ADI) of metals in soil is calculated according to Equations (4)–(6).

(4) $${\mathrm{ADI}}_{\mathrm{ing}}=\frac{C\times {\mathrm{IR}}_{\mathrm{ing}}\times \mathrm{CF}\times \mathrm{EF}\times \mathrm{ED}}{\mathrm{BW}\times \mathrm{AT}}$$

(5) $${\mathrm{ADI}}_{\mathrm{inh}}=\frac{C\times {\mathrm{IR}}_{inh}\times \mathrm{EF}\times \mathrm{ED}}{\mathrm{PEF}\times \mathrm{BW}\times \mathrm{AT}}$$

(6) $${\mathrm{ADI}}_{\mathrm{derm}}=\frac{C\times \mathrm{SA}\times \mathrm{CF}\times \mathrm{SL}\times \mathrm{ABS}\times \mathrm{EF}\times \mathrm{ED}}{\mathrm{BW}\times \mathrm{AT}}$$

where C is the concentration of a specific metal in soil (mg/kg, obtained in this study); IR_ing_ is the ingestion rate (mg/day), which is 100 mg/day for adults and 200 mg/day for children (obtained from USEPA guidebook [51]); EF is the exposure frequency, i.e., 180 days/year (commonly used in literatures, e.g., [21,22]); ED is the exposure duration, i.e., 24 years for adults and 6 years for children [51]; IR_inh_ is the inhalation rate (m^3^/day), i.e., 14.7 m^3^/day for adults and 7.63 m^3^/day for children [57]; PEF is the dust emission factor (m^3^/kg), i.e., 1.36 × 10^9^ m^3^/kg [57]; SA is the exposed area through dermal contact, i.e., 5700 cm^2^ for adults and 2800 cm^2^ for children [57]; SAF is the adherence factor, i.e., 0.2 mg/cm^2^ [51]; ABS is the dermal absorption factor, i.e., 0.001 for all considered elements [51]; BW is body weight, i.e., 57 kg for adults and 22 kg for children [57]; AT is the average exposure time per year, for noncarcinogens: ED × 365 days and for the carcinogens (As, Cr, and Cd): 70 (lifetime) × 365 days [51].

#### 2.3.2. Noncarcinogenic Risk Assessment

A method provided by the US Environmental Protection Agency was used to evaluate the potential health risk associated with the noncarcinogenic effects of metals in soils [58]. The hazard quotient (HQ) was calculated as the ratio of the ADI and the reference dose (RfD) for a given metal [Equation (8)].

(7) HQ=ADI/RfD

where RfD is the reference dose of the i-th metal (mg/kg day^−1^), as listed in Table S1. This dose is the maximum allowable level of a metal with no harmful effects on human health [59].

The sum of the HQ values of all metals in the soil, HI, was used to assess the overall noncarcinogenic effects posed by multiple metals.

(8) $$\mathrm{HI}=\overset{n}{\underset{i=1}{\sum }}{\mathrm{HQ}}_{i}$$

If the HI value is <1, the exposed individual is unlikely to experience obvious adverse health effects; if the HI value is >1, there could be a risk of noncarcinogenic effects.

#### 2.3.3. Carcinogenic Risk Assessment

Carcinogenic risk is defined as the probability of an individual developing any type of cancer throughout his or her lifetime due to exposure to carcinogenic. For a given metal it is calculated according to Equation (9):

(9) $$\mathrm{CR}=\overset{n}{\underset{i=1}{\sum }}{\mathrm{ADI}}_{i}\times {\mathrm{SF}}_{i}$$

For multiple metals, it is the sum of the CR values of different metals [Equation. (10)]:

(10) $$\mathrm{TCR}=\overset{n}{\underset{j=1}{\sum }}{\mathrm{CR}}_{j}$$

where SF is the carcinogenicity slope factor (per mg/kg-day) as listed in Table S1 [60]. Risks < 1.0 × 10^−6^ are considered ignorable; risks lying between 1.0 × 10^−4^ and 1.0 × 10^−6^ are generally considered acceptable, and those > 1.0 × 10^−4^ imply a lifetime carcinogenic risk.

### 2.4. Statistical Analysis 

Significant differences (*p* < 0.05) were examined according to the results of a one-way analysis of variance (ANOVA) with post-hoc multiple comparisons (Tukey or Tamhane). The normality (Kolmogorov-Smirnov and Shapiro-Wilk tests) and homogeneity of variance (Levene’s test) of the data were examined during the ANOVAs. A rotated principal component analysis (PCA) and Pearson’s correlation coefficients analysis were performed to identify common sources and the similar chemical properties of metals [61,62]. Box plots representing six parameters, namely, the minor limit, first quartile, average, third quartile, higher limit, and extreme values, were elaborated for different parameters. SPSS v. 22.0 (IBM, Chicago, IL, USA) was used to perform the analyses, and the geography information system software ArcGIS 10.1 (Redlands, CA, USA) to obtain geochemical maps of the metals. Previous studies report better performance of Kriging interpolation over IDW with relatively high density of sampling sites, while IDW could be more appropriate in the scenario of irregular distribution of sites [63,64,65,66]. Sampling sites in this study were not uniformly distributed, because of the irregular distribution of parks, and residential areas in Suzhou city. Besides, the density of sampling sites was relatively low (i.e., averagely 0.075/km^2^). Inverse distance weighting (IDW) and the natural neighbor module were applied to interpolate the soil metal concentrations to produce the geochemical distribution maps. Results of Kriging interpolation were also depicted in Supporting Information (Figures S3–S5).Uncertainty analysis was processed using Crystal Ball (11.1.2, Oracle, Redwood, CA, USA), with Monte Carlo simulations.

## 3. Results and Discussion

### 3.1. Metal Concentrations in Soils

The concentrations of metals in Suzhou soils and the background reference values in Jiangsu Province are presented in Table 2, together with the enrichment factors (EFs). Our data suggest elevated levels of metals in urban soils of Suzhou city. The average concentrations of As (15.51 mg/kg), Pb (40.26 mg/kg), Hg (0.52 mg/kg), and Cd (0.33 mg/kg) in soils were higher than the background values of Jiangsu, except for Cr (76.6 mg/kg). The EF of Cd (i.e., 2.54) indicated moderate pollution from anthropogenic sources, that of Cr (0.97) slight pollution, and all other values (between 1 and 2) slight pollution by natural or anthropogenic sources [67]. The coefficients of variation (CVs) of metals in urban soils were in the following order: Cd (193.94%) > Pb (79.71%) > Hg (63.38%) > As (41.81%) > Cr (34.79%). The CV for Cd was the highest, indicating high variability and potential impacts of human activities; conversely, the low variability of As and Cr reflected minor impacts of human activities [68].

Compared with the reported metal concentrations of soils from other cities in China (Table S2), the averaged As and Hg concentrations of Suzhou soils were among the highest, whereas the Pb level was exceeded only by that in Nanjing (107.3 mg/kg). Concentration of Cr was second only to that in Shanghai (87.72 mg/kg). However, the averaged Cd concentration was much lower than that in Changsha (6.9 mk/kg), which is heavily polluted by Cd. Thus, it can be concluded that metals contamination of Suzhou soils ranks top among the metropolis in China except few severely polluted ones in southern or southwestern China.

### 3.2. Spatial Distribution of Metals in Different Types of Soils 

As shown in Table 2, the Pb and Cd concentrations of roadside soils were 8−17% or 40−250%, but not significantly higher than those of residential and park soils, while the As, Hg, and Cr concentrations of the three type of soils were comparable. These results suggest potential contribution of traffic to Pb and Cd contamination in roadside soils. In fact, traffic is generally believed to be the major source of Pb and Cd in soils [30].

Generally speaking, concentrations of metals were less variable among districts (Table 3), i.e., maximally 69%, 71%, 48%, 92% and 1% different among districts for As, Pb, Hg, Cr and Cd, respectively. Specifically, Arsenic concentrations in urban soils were significantly higher in Gusu district than in Wuzhong, Xiangcheng, and Yuanqu (*p* < 0.05). By contrast, the Pb and Cr concentrations of the soils in the five districts did not significantly differ (*p* > 0.05). The Hg levels in Xinqu district soils significantly exceeded that in Xiangcheng district soils (*p* < 0.05); the Cd concentrations in Wuzhong district soils were significantly (*p* < 0.05) higher than those of the Yuanqu district. 

**Figure 2:**
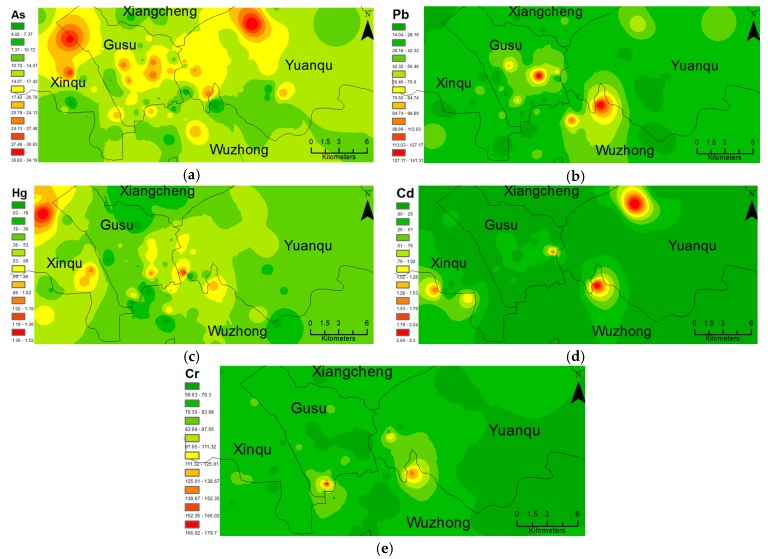
The spatial variation of As (**a**), Pb (**b**), Hg (**c**), Cd (**d**), Cr (**e**) pollution in the soils of Suzhou.

GIS interpolation reveals continuous spatial distribution of the examined metals in Suzhou urban soils (Figure 2). A high As concentration was found in soils from the northeast of Xinqu district, the Gusu district, and the northwest of Yuanqu district; a high Pb concentration in soils from the middle of the Gusu district and the northeast Wuzhong district; a high Hg concentration in soils from the northern Xinqu district and from the junction of the Gusu, Wuzhong, and Yuanqu districts; a high Cd concentration in soils from the northeast Wuzhong and northwest Yuanqu districts; and a high Cr concentration in soils from the northeast Wuzhong and southwest Gusu districts. All these distribution of high polluted zone showed a punctate distribution without continuity, which can be explained by point sources of pollution. Since all the sampling sites were in or not far from residential zones, the point sources of pollution were presumably soils contaminated by plants or factories that had since moved to other suburban areas.

### 3.3. Statistics and Source Analysis 

PCA and correlation analysis were carried out to elucidate the relationships between metals and to identify potential sources of metal pollution in urban soils. As shown in Table 4, three principal components (PCs) explained 76% of the total variation. The first principal component (PC1) accounted for 32% of the total variation and mainly included As, Pb, and Cd, whose variance values were 0.558, 0.827, and 0.736, respectively. PC2 accounted for 24% of the total variation and mainly included Cr, with a variance value of 0.844. PC3 accounted for 21% of the total variation and mainly included Hg, with a variance value of 0.978. The correlation matrix indicates that As significantly and positively correlated with Pb, and Pb with Cd (correlation coefficients of 0.422 and 0.358, respectively; *p* < 0.01 each). The correlation coefficient between As and Cr was −0.184 and that between Pb and Hg 0.170; both relationships were significant at the *p* < 0.05 level.

The results of the PCA were in accordance with those of the correlation analysis. The significant correlations between some of the metals may suggest their common origin [3,69,70]. Thus, As, Pb and Cd likely originated from sources such as battery manufacturing and the dye industry. The sources of Cr, e.g., stainless steel metallurgy and leather tanning, differed from those of Hg, which was probably light bulb manufacturing.

### 3.4. Ecological Risk Assessment of Metals in Urban Soils

Two categories of ecological risk indices, including geo-accumulation index (I_geo_, used for assessing risk of a specific metal) and potential ecological risk index (RI, used for assessing overall risk of different metals), were calculated using Equations (1)–(3) (Table S3) to assess ecological risk of metals in Suzhou urban soils.

The averaged values of I_geo_ for all examined metals were <0, suggesting generally low ecological risk of a specific metal in soils. Most samples, i.e., 56.29% for As, 70.06% for Pb, 54.49% for Hg, 98.20% for Cr, and 79.64% for Cd, showed no evidence of pollution, while 41.32%, 24.55%, 29.94%, 1.2%, and 13.17% of the samples were slightly polluted by As, Pb, Hg, Cr, and Cd. Small percentages of samples were considered moderate pollution, i.e., 2.40% for As, 2.99% for Pb, 14.97% for Hg, 0.60% for Cr, and 4.19% for Cd. Moderate to heavy pollution was identified for 2.4% of samples for Pb, 0.6% for Hg, and 1.2% for Cd, while only 0.6% of samples were found to be heavily polluted by Cd.

**Figure 3:**
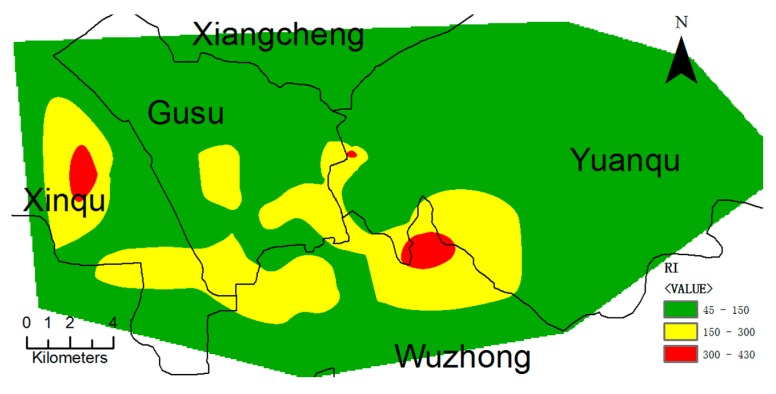
Spatial Distribution of the risk index (RI) in Suzhou soils.

Er and RI (sum of Er for different metals) were calculated, in which both enrichment and toxicity of metals were considered [41]). Similar to I_geo_, average Er values of different metals were generally low (<40, indicating low ecological risk), with the exception of Hg (averagely 70.63, suggesting moderate risk). The average RI of 131.73 implies the low overall ecological risk of metals in Suzhou urban soils. According to the frequency distribution of the RI values, a low, moderate, high, and serious ecological risk was identified in 73.65%, 22.16%, 2.40%, and 1.80% of the samples, respectively. The spatial distribution of the RI (Figure 3) indicates two areas of high ecological risk, i.e., the Xinqu and Wuzhong districts. Xinqu, Wuzhong, and Gusu districts were of moderate ecological, while the other areas were of low ecological risk, as indicated by green in Figure 3. In the two areas with high ecological risk, high concentrations of Hg and Cd in the soils contributed more than 90% to RI values.

### 3.5. Health Risk Assessment of Metals in Soils

**Figure 4:**
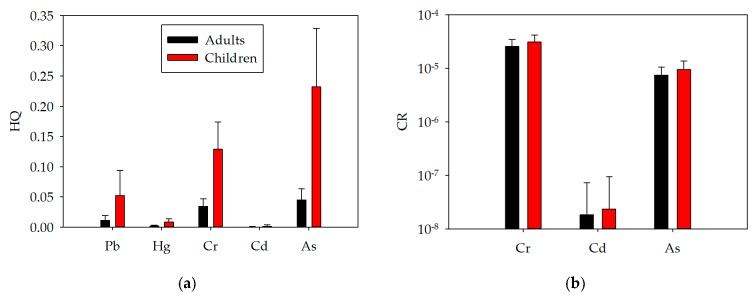
Hazard Quotients (HQs) (**a**) and carcinogenic risks (CRs) (**b**) of the tested metals.

HQ and CR were calculated respectively, to assess the noncarcinogenic risk and carcinogenic risk of a specific metal. The HQ values for all examined metals were lower than 1 (Figure 4a), suggesting low health risk for human exposure to a specific metal in urban soils. The average CR values for adults exposed to Cr, Cd, and As were 2.57 × 10^–5^, 1.82 × 10^–8^ and 7.45 × 10^–6^, respectively, and for children, 3.08 × 10^–5^, 2.35 × 10^–8^ and 9.56 × 10^–6^, respectively (Figure 4b). The CR values of Cd were consistently less than 1.0 × 10^−6^, indicating a negligible carcinogenic risk. For Cr or As, the values were above 1.0 × 10^−6^ or below 1.0 × 10^−4^, and the carcinogenic risk was thus acceptable.

The results of the overall noncarcinogenic risk (assessed by summed values of HQ, i.e., HI) and total carcinogenic risk (assessed by summed values of CR, i.e., TCR) of all metals in soil samples are listed in Table 5. The averaged HI values for both adults and children were far less than 1, indicative of low health risks for people exposed to the metals in Suzhou soils. Only 2.4% of the samples had HI values > 1 (for children only), suggesting a noncarcinogenic risk for children only in extreme cases of exposure. For all examined samples, the TCR values were in the range of 1.0 × 10^−6^ to 1.0 × 10^−4^, except one sample with TCR > 1.0 × 10^−4^. These values are considered acceptable according to literatures [71,72], although higher than the carcinogenic target risk of 1. 0 × 10^−6^ (USEPA, 2011). It is obvious that both noncarcinogenic and carcinogenic risks of metals through ingestion are more than the other two pathway and risks through inhalation are much lower than others, which is same as the other studies on metals risk in soil [53,54].

**Figure 5:**
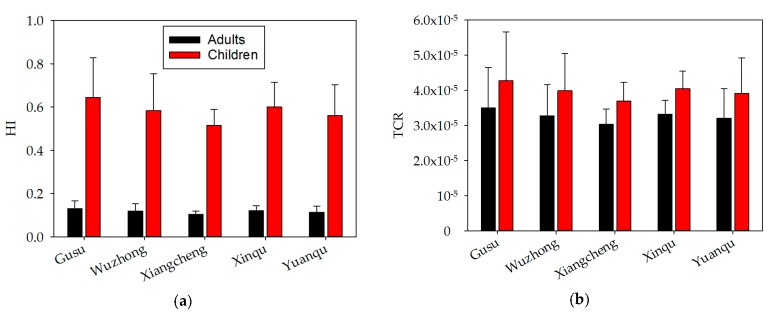
Hazard indices (HIs) (**a**) and total carcinogenic risks (TCRs) (**b**) of the metals in soil samples according to the sampled districts.

HI and TCR values of metals in soil samples of different districts were shown in Figure 5. Both HI and TCR values of metals in the five districts were ranked in the following order: Gusu > Xinqu > Wuzhong > Yuanqu > Xiangcheng, without significant differences among districts. The HI values in the five districts were always <1, indicating low health risk, whereas the TCR values were all in the range of 1.0 × 10^−6^ and 1.0 × 10^−4^, and therefore considered acceptable. 

**Figure 6:**
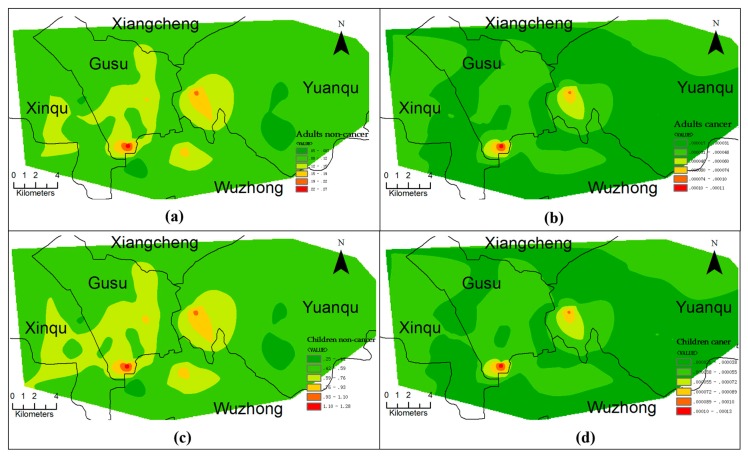
Spatial distribution of Hazard indices (HIs) and total carcinogenic risks (TCRs) for adults (**a**,**b**, respectively) and children (**c**,**d**, respectively).

The spatial distributions of HI and TCR for adults were similar to those for children (Figure 6). For noncarcinogenic risk, only in one site in the southwest of the Gusu district (shown in red in Figure 6a,c), the sample had an HI value more than 1, indicating possible noncarcinogenic risk; in the remaining sites (shown in yellow and green in Figure 6a,c) the noncarcinogenic risks were negligible. The spatial distribution of TCR indicated no risk posed by the soils for Xiangcheng, Xinqu, Wuzhong districts and most area of Gusu and Yuanqu districts (shown in green in Figure 6b,d), an acceptable risk in other area of Gusu and Yuanqu districts (shown in yellow in Figure 6b,d), and the point with the highest HI also had the highest TCR (>1.0 × 10^−4^) (shown in red in Figure 6b,d).

### 3.6. Uncertainty Analysis of the Risk Assessments

**Figure 7:**
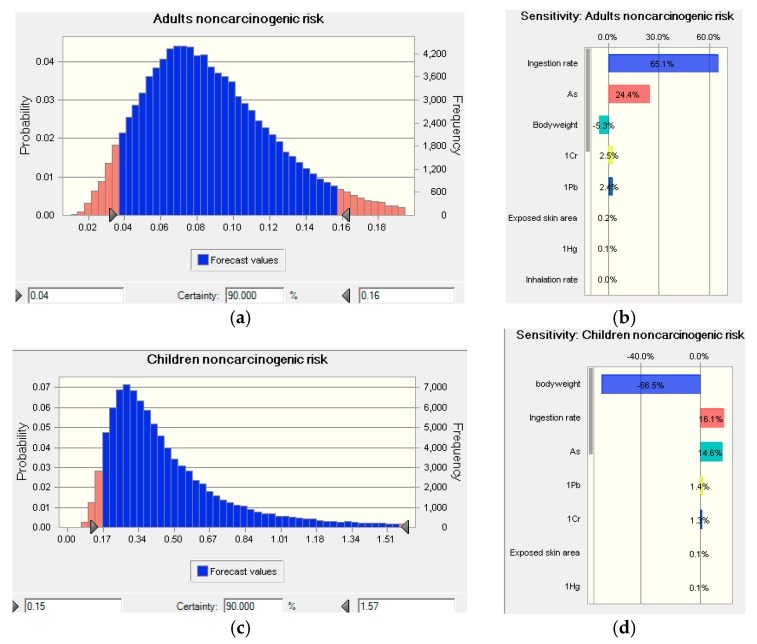

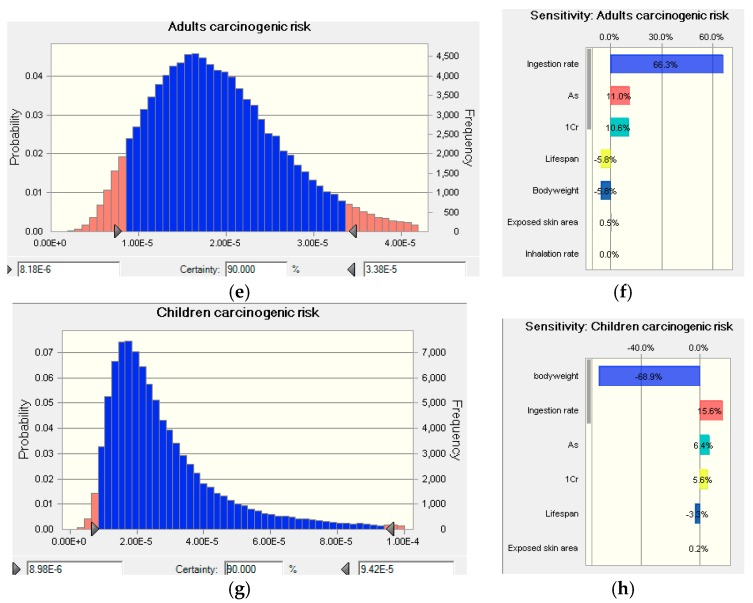
Probability distribution of the noncarcinogenic (**a**–**d**) and carcinogenic (**e**–**h**) health risks of metals and the uncertainty analysis for adults (**a**,**b**,**e**,**f**) and children (**c**,**d**,**g**,**h**).

The concentrations of the five metals were entered as input into the Crystal Ball software for probability distribution fitting, which matches each value against each continuous probability distribution and chooses the distribution with the highest-ranking fit to represent the data. The results are shown in Table S4 and Figure S2. The values of As, Hg, Cd, Cr, and Pb concentrations were fitted to the max extreme, gamma, lognormal, Student’s t, and log-normal distributions. For the other parameters, data were collected from the literatures [58,73,74,75], and are listed in Table S5. The values of body weight and lifetime were normally distributed, while those of the ingestion rate, inhalation rate, and exposed skin area followed a triangular distribution. All of these uncertain parameters were imported into the Crystal Ball software for stochastic simulation, which then determined the probability of the health risk and the sensitivity of the selected parameter (Figure 7). 

For adults, the 5% percentile, median, mean, and 95% percentile of HI were 0.04, 0.08, 0.09, and 0.16; the corresponding values for children were 0.15, 0.39, 0.57, and 1.57, respectively. The 5% percentile, median, mean, and 95% percentile of the TCR for adults were 8.18 × 10^–6^, 1.82 × 10^–5^, 1.93 × 10^–5^, and 3.38 × 10^–5^, and for children 8.98 × 10^–6^, 2.34 × 10^–5^, 3.47 × 10^–5^, and 9.42 × 10^–5^, respectively. Most of these results were lower than those obtained from models using deterministic parameters (listed in Table 4). Given that all the deterministic parameters adopted average values, which were unable to take all situations into consideration, it may be implied that the results from models using deterministic parameters overestimated the health risks associated with metal in Suzhou soils.

Differences between adults and children were clearly shown in the sensitivity analysis. For adults, for both HI and TCR, the ingestion rate contributed >60% to the sensitivity of the parameter, which is accordant with the risk assessment result of three pathway above. And the soil As concentration ≥10%. Other parameters (body weight, lifetime, Cr and Pb concentrations, etc.) contributed <10%. For children, body weight was the largest contributor (66–68%), followed by the ingestion rate (15–16%), and As concentration (6.4–14.6%). Lifetime exposure, as well as Pb and Cr concentrations, accounted for the remainder (0.2–5.6%). These results suggest that adults can lower the health risk from soil-associated metals by reducing intake via oral pathway, while body weight is a key determinant of the health risks for children.

## 4. Conclusions

This study determined the concentrations, spatial distribution, ecological risk, and health risk of metals in urban soils from a typical industrial city, Suzhou, Eastern China. The mean concentrations of As, Cd, Pb, and Hg, but not Cr, were higher than the background values in Jiangsu Province. The concentrations of metals were moderately variable among districts and sampling sites but overall a punctate distribution was observed. The PCA and correlation analysis revealed that As, Pb, and Cd originated from the same sources. Results of risk assessment suggest ecological and health risks of metals in urban soils in Suzhou: The I_geo_ and RI showed the slight contamination of urban soils in Suzhou city. Analysis of HQ indicates that the levels of As, Pb, Hg, Cr and Cd were relatively safe for Suzhou residents, and the CRs of Cr and As were in the range of 1. 0 × 10^−6^ and 1.0 × 10^−4^, suggesting acceptable risks. Among the uncertain parameters considered in the health risk model, the ingestion rate and body weight were the most sensitive for adults and children, respectively, with As being an important contributor for both. Results gained in this study could help better understand the risks of metals in urban soils of industrial cities in China. 

## Figures and Tables

**Table 1:** Classification of I_geo_ and RI.

Type	Range	Level	Type	Range	Level	Type	Range	Level
I_geo_	I_geo_ < 0	unpolluted	E_r_	E_r_ < 40	Low	RI	RI < 150	Low
	0 ≤ I_geo_ < 1	unpolluted to moderately polluted		40 ≤ E_r_ < 80	Moderate		150 ≤ RI < 300	Moderate
	1 ≤ I_geo_ < 2	moderately polluted		80 ≤ E_r_ < 160	High		300 ≤ RI < 600	High
	2 ≤ I_geo_ < 3	moderately to heavily polluted		160 ≤ E_r_ < 320	serious		RI ≥ 600	serious
	3 ≤ I_geo_ < 4	heavily polluted		E_r_ ≥ 320	severe			
	4 ≤ I_geo_ < 5	heavily to extremely polluted						
	I_geo_ ≥ 5	extremely polluted						

**Table 2:** Statistical characteristics of the metal concentrations of Suzhou soils.

Name	As	Pb	Hg	Cr	Cd
Range (mg/kg)	1.10−55.46	8.80−243.00	0−1.81	46.20−299.95	0−4.80
Mean ± SD (mg/kg)	15.51 ± 6.48	40.26 ± 32.09	0.52 ± 0.34	75.60 ± 26.30	0.33 ± 0.64
Jiangsu background level (mg/kg)	10	26.2	0.29	77.8	0.13
CV (%)	41.81	79.71	65.38	34.79	193.94
EF	1.55	1.54	1.79	0.97	2.54

SD = standard deviation; CV = coefficient of variation; EF = enrichment factor.

**Table 3:** Distribution characteristics of soil metals in different land use types and different districts.

Metals (mg/kg)	As	Pb	Hg	Cr	Cd
			Roadside		
Range	4.00−34.20	4.86−243.00	0.03−1.81	46.18−217.82	0.001−4.80
Mean ± SD	16.20 ± 6.08	42.22 ± 38.34	0.51 ± 0.38	75.23 ± 20.96	0.21 ± 0.64
			Resident		
Range	4.41−55.46	8.82−184.00	0.03−1.51	51.93−299.95	0.001−2.31
Mean ± SD	15.30 ± 7.24	39.21 ± 31.06	0.51 ± 0.34	74.92 ± 29.81	0.15 ± 0.34
			Park		
Range	1.13−24.38	15.00−87.87	0.10−1.24	49.90−221.61	0.001−0.48
Mean ± SD	14.45 ± 5.50	35.99 ± 15.60	0.52 ± 0.29	77.92 ± 29.22	0.06 ± 0.11
			Gusu District		
Range	9.58−55.46	4.86−222.00	0.06−1.22	51.18−299.95	0.001−0.90
Mean ± SD	17.66 ± 7.22 ^a^	45.28 ± 37.57	0.50 ± 0.32	77.94 ± 34.78	0.15 ± 0.21
			Wuzhong District		
Range	4.00−24.05	5.25−243.00	0.03−1.45	52.85−217.82	0.002−4.80
Mean ± SD	13.58 ± 5.49 ^b^	47.71 ± 49.35	0.52 ± 0.39	77.09 ± 28.62	0.34 ± 0.95 ^e^
			Xiangcheng District		
Range	9.75−16.62	24.58−48.10	0.22−0.44	63.89−97.13	0.001−0.005
Mean ± SD	12.15 ± 2.43 ^b^	36.11 ± 9.48	0.30 ± 0.07 ^c^	72.21 ± 10.98	0.003 ± 0.001
			Xinqu District		
Range	5.57−31.76	13.82−59.96	0.18−1.49	54.48−89.59	0.002−1.27
Mean ± SD	16.64 ± 6.53	34.54 ± 13.14	0.62 ± 0.35 ^d^	74.05 ± 9.12	0.19 ± 0.25
			Yuanqu District		
Range	1.13−34.20	10.27−147.00	0.05−1.81	46.18−221.61	0.001−2.31
Mean ± SD	14.52 ± 6.10 ^b^	33.88 ± 21.38	0.49 ± 0.35	73.96 ± 23.86	0.07 ± 0.32 ^f^

Significant differences were shown between ^a^ and ^b^, ^c^ and ^d^, ^e^ and ^f^ (*p* < 0.05).

**Table 4:** Principal component analysis (PCA) and correlation coefficients of metals in Suzhou soils.

PCA				Pearson‘s Correlation Coefficient					
Metal	PC1	PC2	PC3	Metal	As	Pb	Hg	Cr	Cd
As	0.558	−0.632	0.130	As	1				
Pb	0.827	−0.093	0.199	Pb	0.422 **	1			
Hg	0.051	0.017	0.978	Hg	0.101	0.170 *	1		
Cr	0.202	0.844	0.067	Cr	−0.184 *	0.100	0.012	1	
Cd	0.736	0.254	−0.133	Cd	0.108	0.358 **	0.036	0.122	1
Eigenvalue	1.665	1.176	0.962						
Variance/%	31.63	23.72	20.69						
Cumulative/%	31.63	55.35	76.04						

** Correlation is significant at the 0.01 level (2-tailed); * Correlation is significant at the 0.05 level (2-tailed).

**Table 5:** Health risk assessment of metals in soils.

	HI		TCR	
	Adults	Children	Adults	Children
Minimum	0.0510	0.2473	1.75 × 10^–5^	2.10 × 10^–5^
5th percentile	0.0860	0.4248	2.49 × 10^–5^	3.03 × 10^–5^
Mean	0.1199	0.5935	3.31 × 10^–5^	4.04 × 10^–5^
Median	0.1147	0.5632	3.19 × 10^–5^	3.89 × 10^–5^
95th percentile	0.1699	0.8499	4.12 × 10^–5^	5.01 × 10^–5^
Maximum	0.2750	1.3746	1.08 × 10^–5^	1.30 × 10^–5^
Standard deviation	0.0315	0.1572	8.91 × 10^–5^	1.07 × 10^–5^
Ingestion pathway	0.0780	0.4041	1.81 × 10^–5^	2.35 × 10^–5^
Inhalation pathway	0.0003	0.0003	1.07 × 10^–7^	3.59 × 10^–8^
Dermatic pathway	0.0417	0.1890	1.49 × 10^–5^	1.69 × 10^–5^

HI, hazard index; TCR, total carcinogenic risk.

## Supplementary Materials

The following are available online at www.mdpi.com/1660-4601/14/9/1025/s1, Figure S1: Time course of the publication of articles on the risk posed by metals in soil (a), urban soil (b), farmland (c), and industrial land (d). Figure S2: Probability distribution fitting of the soil concentration of As (a), Hg (b), Cr (c), Cd (d), and Pb (e). Table S1: Summary of the reference dose (RfD) and cancer slope factor (SF) of the metals. Table S2: Comparison of the background values in Jiangsu and other cities. Table S3: Ecological risk assessment of metals in soil samples. Table S4: Probability distribution fitting of the metal concentrations. Table S5: Probability distribution of the parameters used in the health risk assessment.

## References

[1] Ministry of Environmental Protection of China, Ministry of Land and Resources of China (2014). The Report on National General Survey on Soil Contamination.

[2] De Miguel E., Iribarren I., Chacon E., Ordonez A., Charlesworth S. (2007). Risk-based evaluation of the exposure of children to trace elements in playgrounds in Madrid (Spain). Chemosphere.

[3] Qu C.S., Ma Z.W., Yang J., Liu Y., Bi J., Huang L. (2012). Human exposure pathways of heavy metals in a lead-zinc mining area, Jiangsu Province, China. PLoS ONE.

[4] Cao S., Duan X., Zhao X., Chen Y., Wang B., Sun C., Zheng B., Wei F. (2016). Health risks of children‘s cumulative and aggregative exposure to metals and metalloids in a typical urban environment in China. Chemosphere.

[5] Khan S., Munir S., Sajjad M., Li G. (2016). Urban park soil contamination by potentially harmful elements and human health risk in Peshawar city, Khyber Pakhtunkhwa, Pakistan. J. Geochem. Explor..

[6] Oberdorster G., Gelein R.M., Ferin J., Weiss B. (1995). Association of particulate air pollution and acute mortality: Involvement of ultrafine particles. Inhal. Toxicol..

[7] Liu W.H., Zhao J.Z., Ouyang Z.Y., Soderlund L., Liu G.H. (2005). Impacts of sewage irrigation on heavy metal distribution and contamination in Beijing, China. Environ. Int..

[8] Wong C.S., Li X., Thornton I. (2006). Urban environmental geochemistry of trace metals. Environ. Pollut..

[9] Cabral Pinto M., Silva M., Silva E., Dinis P., Rocha F. (2017). Transfer processes of potentially toxic elements (PTE) from rocks to soils and the origin of PTE in soils: A case study on the island of Santiago (Cape Verde). J. Geochem. Explor..

[10] Cabral Pinto M.M.S., da Silva E.F., Silva M.M.V.G., Melo-Goncalves P. (2015). Heavy metals of Santiago island (Cape Verde) top soils: Estimated background value maps and environmental risk assessment. J. Afr. Earth Sci..

[11] Pinto M., Silva E., Silva M., Melo-Gonçalves P., Candeias C. (2014). Environmental risk assessment based on high-resolution spatial maps of potentially toxic elements sampled on stream sediments of Santiago, Cape Verde. Geosciences.

[12] Smith S.R. (2009). A critical review of the bioavailability and impacts of heavy metals in municipal solid waste composts compared to sewage sludge. Environ. Int..

[13] Hamzeh M.A., Aftabi A., Mirzaee M. (2011). Assessing geochemical influence of traffic and other vehicle-related activities on heavy metal contamination in urban soils of Kerman city, using a GIS-based approach. Environ. Geochem. Health.

[14] Li X., Liu L., Wang Y., Luo G., Chen X., Yang X., Hall M.H.P., Guo R., Wang H., Cui J. (2013). Heavy metal contamination of urban soil in an old industrial city (Shenyang) in Northeast China. Geoderma.

[15] Xia X.H., Chen X., Liu R.M., Liu H. (2011). Heavy metals in urban soils with various types of land use in Beijing, China. J. Hazard. Mater..

[16] Liu D.X., Li Y.M., Ma J.H., Li C., Chen X. (2016). Heavy metal pollution in urban soil from 1994 to 2012 in Kaifeng city, China. Water Air Soil Poll..

[17] Liu Q., Liu J.S., Wang Q.C., Wang Y. (2015). Assessment of heavy metal pollution in urban agricultural soils of Jilin city, China. Hum. Ecol. Risk Assess..

[18] Wang X.S. (2014). Heavy metal geochemistry and mineral magnetic characterization of urban soil in Xuzhou, China. Environ. Earth Sci..

[19] Cheng H.X., Li M., Zhao C.D., Li K., Peng M., Qin A.H., Cheng X.M. (2014). Overview of trace metals in the urban soil of 31 metropolises in China. J. Geochem. Explor..

[20] Cai Q.Y., Mo C.H., Li H.Q., Lu H.X., Zeng Q.Y., Li Y.W., Wu X.L. (2013). Heavy metal contamination of urban soils and dusts in Guangzhou, South China. Environl. Monit. Assess..

[21] Zhao L., Xu Y.F., Hou H., Shangguan Y.X., Li F.S. (2014). Source identification and health risk assessment of metals in urban soils around the tanggu chemical industrial district, Tianjin, China. Sci. Total Environ..

[22] Pan L., Ma J., Hu Y., Su B., Fang G., Wang Y., Wang Z., Wang L., Xiang B. (2016). Assessments of levels, potential ecological risk, and human health risk of heavy metals in the soils from a typical county in Shanxi Province, China. Environ. Sci. Pollut. Res..

[23] Chen X., Xia X., Zhao Y., Zhang P. (2010). Heavy metal concentrations in roadside soils and correlation with urban traffic in Beijing, China. J. Hazard. Mater..

[24] Wang G., Zeng C., Zhang F., Zhang Y., Scott C.A., Yan X. (2017). Traffic-related trace elements in soils along six highway segments on the Tibetan Plateau: Influence factors and spatial variation. Sci. Total Environ..

[25] Christoforidis A., Stamatis N. (2009). Heavy metal contamination in street dust and roadside soil along the major national road in Kavala’s region, Greece. Geoderma.

[26] De Silva S., Ball A.S., Huynh T., Reichman S.M. (2016). Metal accumulation in roadside soil in Melbourne, Australia: Effect of road age, traffic density and vehicular speed. Environl. Pollut..

[27] Lei J., Hasi E., Sun Y. (2015). Assessing the influence of different road traffic on heavy metal accumulation in rural roadside surface soils of the eastern ordos plateau grassland in China. Water Resources and Environment.

[28] Qiao X., Schmidt A.H., Tang Y., Xu Y., Zhang C. (2014). Demonstrating urban pollution using toxic metals of road dust and roadside soil in Chengdu, Southwestern China. Stoch. Environ. Res. Risk A.

[29] Padoan E., Rome C., Ajmone-Marsan F. (2017). Bioaccessibility and size distribution of metals in road dust and roadside soils along a peri-urban transect. Sci. Total Environ..

[30] Werkenthin M., Kluge B., Wessolek G. (2014). Metals in european roadside soils and soil solution—A review. Environl. Pollut..

[31] Yang J., Teng Y.G., Song L.T., Zuo R. (2016). Tracing sources and contamination assessments of heavy metals in road and foliar dusts in a typical mining city, China. PLoS ONE.

[32] Nezat C.A., Hatch S.A., Uecker T. (2017). Heavy metal content in urban residential and park soils: A case study in Spokane, Washington, USA. Appl. Geochem..

[33] Figueiredo A.M.G., Enzweiler J., Camargo S.P., Sigolo J.B., Gumiero F.C., Pavese A.C., Milian F.M. (2009). Metal contamination in urban park soils of SA o pound Paulo. J. Radioanal. Nucl. Chem..

[34] Mazurek R., Kowalska J., Gąsiorek M., Zadrożny P., Józefowska A., Zaleski T., Kępka W., Tymczuk M., Orłowska K. (2017). Assessment of heavy metals contamination in surface layers of Roztocze National Park Forest soils (SE Poland) by indices of pollution. Chemosphere.

[35] Liu J., Yang J., Luo W., Ke L., Huang P., Xiang B. (2012). Effect of heavy metals on brownfield quality in different industries. Adv. Mater. Res..

[36] Sharma K., Basta N.T., Grewal P.S. (2015). Soil heavy metal contamination in residential neighborhoods in post-industrial cities and its potential human exposure risk. Urban Ecosyst..

[37] Rodriguez-Seijo A., Andrade M.L., Vega F.A. (2017). Origin and spatial distribution of metals in urban soils. J. Soil Sediment.

[38] Kuzmanoski M.M., Todorovic M.N., Urosevic M.P.A., Rajsic S.F. (2014). Heavy metal content of soil in urban parks of Belgrade. Hemijska Ind..

[39] Förstner U., Ahlf W., Calmano W. (1993). Sediment quality objectives and criteria development in Germany. Water Sci. Technol..

[40] Ji Y., Feng Y., Wu H., Zhu T., Bai Z., Duan C. (2008). Using geoaccumulation index to study source profiles of soil dust in china. J. Environ. Sci..

[41] Hakanson L. (1980). An ecological risk index for aquatic pollution control. A sedimentological approach. Water Res..

[42] Bai Y., Wang M., Peng C., Alatalo J.M. (2016). Impacts of urbanization on the distribution of heavy metals in soils along the Huangpu River, the drinking water source for Shanghai. Environ. Sci. Pollut. Res..

[43] Duan X., Zhang G., Rong L., Fang H., He D., Feng D. (2015). Spatial distribution and environmental factors of catchment-scale soil heavy metal contamination in the dry-hot valley of upper red river in Southwestern China. Catena.

[44] Qu L., Xie Y., Lu G., Yang C., Zhou J., Yi X., Dang Z. (2017). Distribution, fractionation, and contamination assessment of heavy metals in paddy soil related to acid mine drainage. Paddy Water Environ..

[45] Ministry of Environmental Protection of China (1995). Environmental Quality Standard for Soils (GB 15618-1995).

[46] Muller G. (1969). Index of geoaccumulation in sediments of the Rhine river. GEO J..

[47] Müller G. (1971). Schwermetalle in Den Sedimenten Des Rheins-Veräderungen Seit 1971. Umschau Verlag,.

[48] Wei F.S., Chen J.S. (1991). Research on the background values of soil in China. Environ. Sci..

[49] Dauvalter V., Rognerud S. (2001). Heavy metal pollution in sediments of the Pasvik river drainage. Chemosphere.

[50] Maanan M., Saddik M., Maanan M., Chaibi M., Assobhei O., Zourarah B. (2015). Environmental and ecological risk assessment of heavy metals in sediments of Nador Lagoon, Morocco. Ecol. Indic..

[51] U.S. Environmental Protection Agency (USEPA) (1997). Exposure Factors Handbook.

[52] Ying L., Shaogang L., Xiaoyang C. (2016). Assessment of heavy metal pollution and human health risk in urban soils of a coal mining city in east China. Hum. Ecol. Risk Assess..

[53] Islam M.S., Ahmed M.K., Habibullah-Al-Mamun M., Eaton D.W. (2017). Human and ecological risks of metals in soils under different land use in an urban environment of Bangladesh. Pedosphere.

[54] Hu B., Wang J., Jin B., Li Y., Shi Z. (2017). Assessment of the potential health risks of heavy metals in soils in a coastal industrial region of the Yangtze River delta. Environ. Sci. Pollut. Res..

[55] Bourliva A., Papadopoulou L., Aidona E., Giouri K., Simeonidis K., Vourlias G. (2017). Characterization and geochemistry of technogenic magnetic particles (TMPS) in contaminated industrial soils: Assessing health risk via ingestion. Geoderma.

[56] Li F., Zhang J., Jiang W., Liu C., Zhang Z., Zhang C., Zeng G. (2017). Spatial health risk assessment and hierarchical risk management for mercury in soils from a typical contaminated site, China. Environ. Geochem. Health.

[57] Duan X. (2013). Highlights of the Chinese Adults' Exposure Factors Handbook.

[58] U.S. Environmental Protection Agency (USEPA) (2002). Supplemental Guidance for Developing Soil Screening Levels for Superfund Sites.

[59] USEPA (1991). Risk Assessment Guidance for Superfund Volume I Human Health Evaluation Manual (Part A) Interim Final.

[60] Ministry of Environmental Protection of China (2014). Technical Guidelines for Risk Assessment of Contaminated Sites.

[61] Arslan H., Ayyildiz Turan N. (2015). Estimation of spatial distribution of heavy metals in groundwater using interpolation methods and multivariate statistical techniques; its suitability for drinking and irrigation purposes in the middle black sea region of Turkey. Environl. Monit. Assess..

[62] Yuan G.L., Sun T.H., Han P., Li J. (2013). Environmental geochemical mapping and multivariate geostatistical analysis of heavy metals in topsoils of a closed steel smelter: Capital iron & steel factory, Beijing, China. J. Geochem. Explor..

[63] Chen Y., Shan X., Jin X., Yang T., Dai F., Yang D. (2016). A comparative study of spatial interpolation methods for determining fishery resources density in the yellow sea. Acta Oceanol. Sin..

[64] Mirzaei R., Sakizadeh M. (2016). Comparison of interpolation methods for the estimation of groundwater contamination in andimeshk-shush plain, southwest of Iran. Environ. Sci. Pollut. Res..

[65] Zhen Z., Guo Z.-Y., Zhao Y.-H., Li F.-R., Wei Q.-B. (2016). Spatial distribution of soil total nitrogen in liangshui national nature reserve based on local model. J. Appl. Ecol..

[66] He Y.H., Lin K.R. (2010). Application and camparison of rainfall spatial interpolation methods on Dongjiang River basin. Water Power.

[67] Gu Y.G., Gao Y.P., Lin Q. (2016). Contamination, bioaccessibility and human health risk of heavy metals in exposed-lawn soils from 28 urban parks in Southern China’s largest city, Guangzhou. App. Geochem..

[68] Li X., Poon C.S., Liu P.S. (2001). Heavy metal contamination of urban soils and street dusts in Hong Kong. Appl. Geochem..

[69] Facchinelli A., Sacchi E., Mallen L. (2001). Multivariate statistical and gis-based approach to identify heavy metal sources in soils. Environ. Pollut..

[70] Mico C., Recatala L., Peris A., Sanchez J. (2006). Assessing heavy metal sources in agricultural soils of an European mediterranean area by multivariate analysis. Chemosphere.

[71] Wu S., Peng S.Q., Zhang X.X., Wu D.L., Luo W., Zhang T.B., Zhou S.G., Yang G.Y., Wan H.F., Wu L.Q. (2015). Levels and health risk assessments of heavy metals in urban soils in Dongguan, China. J. Geochem. Explor..

[72] Guney M., Zagury G.J., Dogan N., Onay T.T. (2010). Exposure assessment and risk characterization from trace elements following soil ingestion by children exposed to playgrounds, parks and picnic areas. J. Hazard. Mater..

[73] Duan X.L., Nie J., Wang Z.S., Wang F.F., Huang N., Che F., Zhang J.L. (2009). Human exposure factors in health risk assessment. J. Environ. Health.

[74] Hou J., Qu Y.H., Ning D.L., Wang H. (2014). Characteristic of human exposure factors in China and their uncertainty analysis in health risk assessment. Environ. Sci. Technol..

[75] Duan X.L. (2014). Highlight of Chinese Children's Exposure Factors Handbook.

