# Supplementary material for: Risk Assessment of Metals in Urban Soils from a Typical Industrial City, Suzhou, Eastern China
Source: Int J Environ Res Public Health. 2017 Sep 7;14(9):1025. doi: 10.3390/ijerph14091025 (PMC5615562; doi:10.3390/ijerph14091025)
Supplement: Supplementary file 1 [file ijerph-14-01025-s001.pdf]

Supplementary Information

Assessment of heavy metal risks in urban soils from a typical industrial city, Suzhou, Eastern China

Gang Wang, Yu Gong, Hou-qi Liu, Ai-Jun Miao, Liu-Yan Yang, Huan Zhong

Table S1. Summary of reference dose (RfD) and cancer slope factor (SF) of heavy metals

item	As	Cd	Cr	Hg	Pb
$RfD_{ing}/mg \cdot (kg \cdot d)^{-1}$	0.0003	0.001	0.003	0.0003	0.0035
$RfD_{inh}/mg \cdot (kg \cdot d)^{-1}$	0.0003	0.001	0.000029	0.0003	0.0035
$RfD_{derm}/mg \cdot (kg \cdot d)^{-1}$	0.00012	0.00001	0.00006	0.000021	0.00052
$SF_{ing}/(kg \cdot d) \cdot mg^{-1}$	1.5	0.38	0.5		
$SF_{inh}/(kg \cdot d) \cdot mg^{-1}$	15	6.1	42		
$SF_{derm}/(kg \cdot d) \cdot mg^{-1}$	3.7	0.38	20.0		

RfD_{ing}, RfD_{inh}, RfD_{derm} and SF_{ing}, SF_{inh}, SF_{derm} indicate RfD and SF via ingestion, inhalation and dermal contact, respectively.

Table S2. Comparison with values of the background in Jiangsu and other cities

	As	Pb	Hg	Cr	Cd
Range	1.10-55.46	8.80-243.00	0-1.81	46.20-299.95	0-4.80
Mean± standard deviation	15.51±6.48	40.26±32.09	0.52±0.34	75.60±26.30	0.33±0.64
Taiyuan [1]	10.96	26.29	0.12	73.69	0.21
Beijing [2]	-	23.30	-	61.00	0.13
Tianjin [3]	11	45	0.43	51	0.18
Changchun [4]	12.5	35.4	0.12	66	0.13
Shanghai [5]	-	28.86	-	87.72	-
Hefei [6]	10.8	37.0	0.18		0.20
Huainan [7]	12.54	24.21	0.21	49.39	0.19
Guangzhou [8]	-	65.40	-	22.40	0.23
Nanjing [9]	-	107.30	-	84.70	-
Changsha [10]		89.4		121	6.9

Table S3. Ecological risk assessment of metals in soil samples

	As	Pb	Hg	Cr	Cd
	geo-accumulation index (I_{geo})				
Mean±SD.	-0.08±0.63	-0.24±0.81	-0.11±1.10	-0.67±0.33	-3.50±3.26
Range	-3.73～1.89	-3.02～2.63	-4.00～2.06	-1.34～1.36	-7.34～4.62
Level 0	56.29 %	70.06 %	54.49 %	98.20 %	79.64 %
Level 1	41.32 %	24.55 %	29.94 %	1.20 %	13.17 %
Level 2	2.40 %	2.99 %	14.97 %	0.60 %	4.19 %
Level 3	0%	2.40 %	0.60 %	0%	1.20 %
Level 4	0%	0%	0%	0%	0.60 %
Level 5	0%	0%	0%	0%	0 %
	potential ecological risk factor (E_r)				
Mean±SD.	15.51±6.48	7.60±6.13	70.63±47.47	1.94±0.68	36.04±107.60
Range	1.13～55.46	0.93～46.37	3.74～249.66	1.19～7.71	0.28～1106.59
Level 1	99.40 %	98.80 %	25.15%	99.40	75.45%
Level 2	0.60 %	1.20 %	44.91%	0.60	15.57%
Level 3	0 %	0 %	22.75%	0 %	4.79%
Level 4	0 %	0 %	7.19%	0 %	2.39%
Level 5	0 %	0 %	0 %	0 %	1.80%
	potential ecological risk index (RI)				
Mean±SD.	131.73±122.95				
Range	26.12～1168.54				
Level 1	73.65%				
Level 2	22.16%				
Level 3	2.40%				
Level 4	1.80%				

Table S4. Probability distribution fitting of heavy metals contents

Parameter	Mean	Median	SD.	Min	Max	Statistical distribution
As content	15.5	14.52	6.22	2.78	39.79	Max Extreme
Hg content	0.51	0.45	0.32	0.01	1.80	Gamma
Cd content	0.22	0.02	2.84	0.00	12.78	Lognormal
Cr content	73.04	72.12	19.08	0.01	291.25	Student's t
Pb content	38.81	33.31	22.92	5.27	188.15	Lognormal

Table S5. Probability distribution of the parameters in health risk assessment

Parameter	Unit	Median	SD.	Min	Max	Statistical distribution
Adults bodyweight	kg	57	5.8	42.1	71.6	Normal
Children bodyweight	kg	22	10.98	5.35	38.15	Normal
Adults ingestion rate	Mg/d	100		20	200	Triangle
Children ingestion rate	Mg/d	200		85	300	Triangle
Adults inhalation rate	M3/d	14.7		6.24	114	Triangle
Children inhalation rate	M3/d	7.63		2	34.32	Triangle
Adults exposed skin area	M2	5700		760	8800	Triangle
Children exposed skin area	M2	2800		430	5200	Triangle
Lifetime	Year	70	7.1			Normal

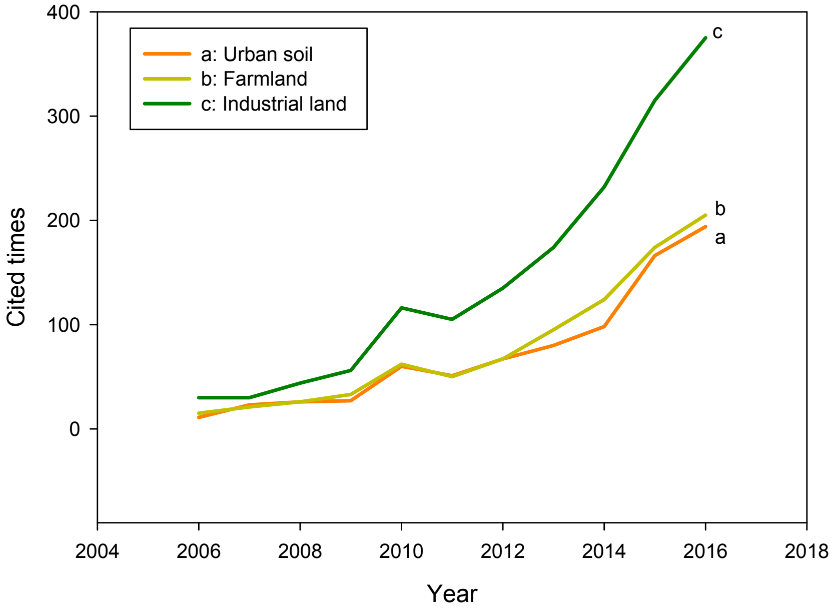

Figure S1. Cited times of the articles on the metal risk in (a) urban soil, in (b) farmland, in (c) industrial land. (Accessed via Web of Science on 25 Jun 2017, keywords for a, b, c are 'soil and metal and risk and china and (urban or city)', 'soil and metal and risk and china and (farm* or cropland or argicult*)', 'soil and metal and risk and china and (industr* or manufact*)').

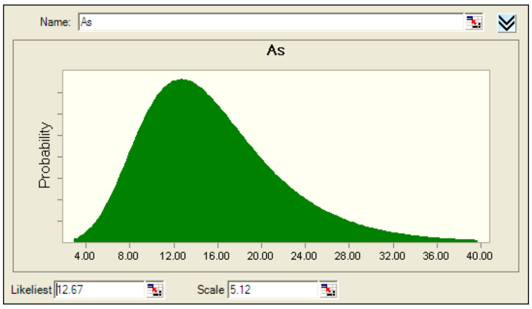

(a)

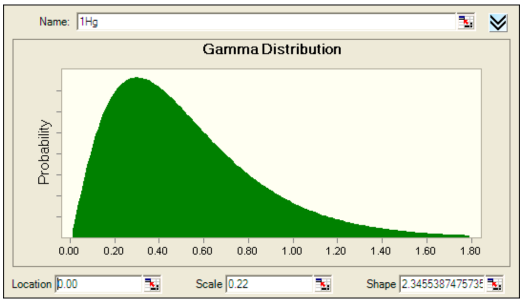

(b)

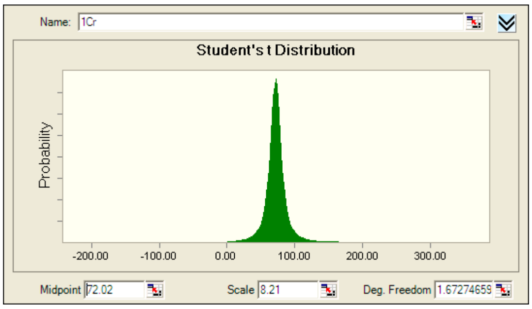

(c)

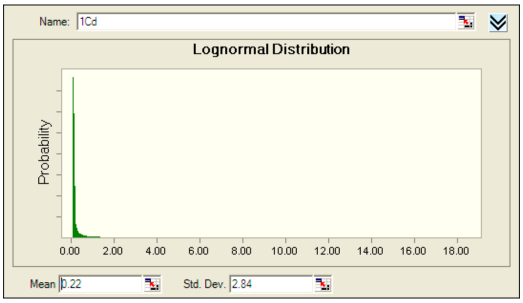

(d)

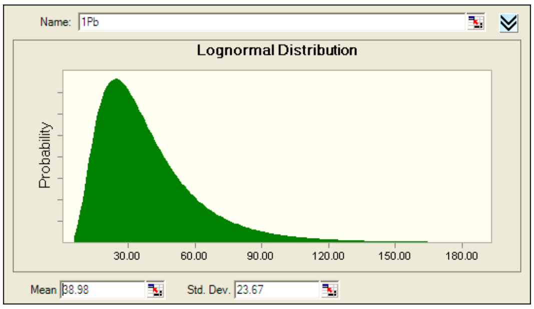

(e)

Figure S2. Probability distribution fitting of contents of As (**a**), Hg (**b**), Cr (**c**), Cd (**d**) and Pb (**e**).

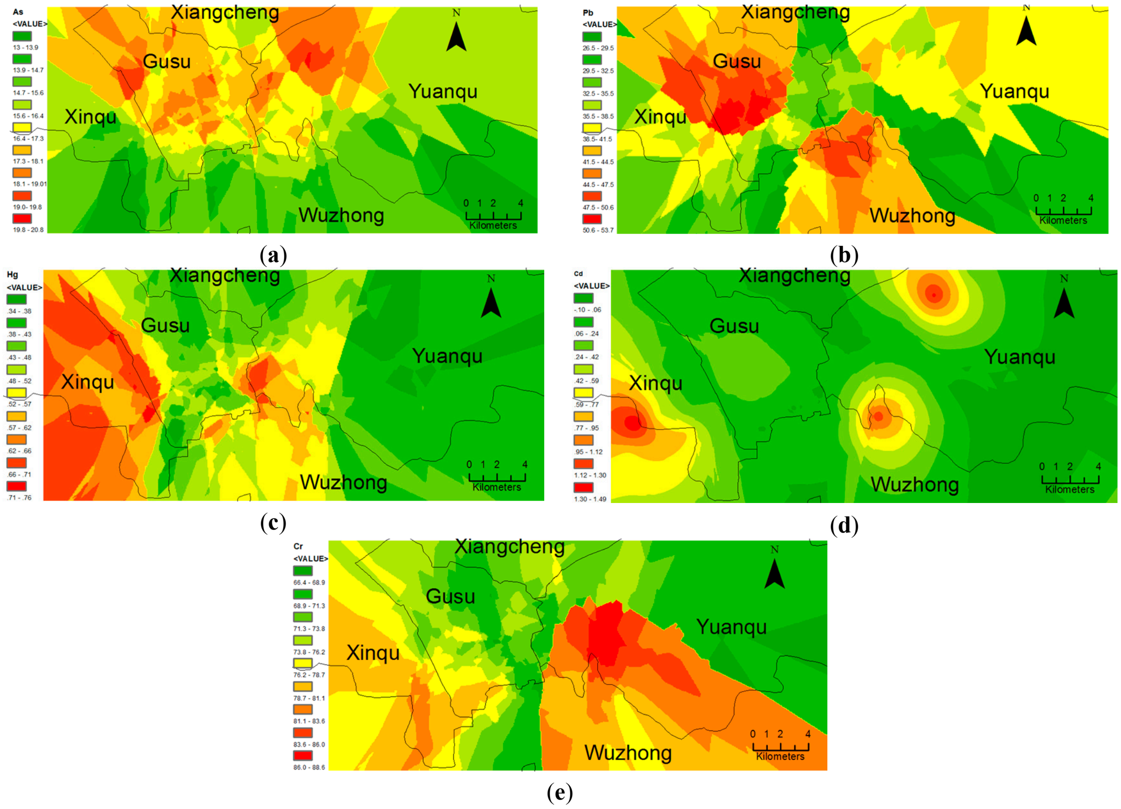

Figure S3. The spatial variation of As (**a**), Pb (**b**), Hg (**c**), Cd (**d**), Cr (**e**) pollution in the soils of Suzhou by Kriging interpolation

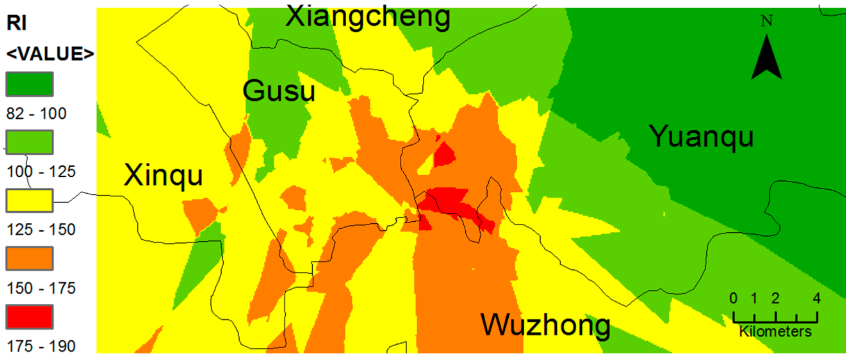

Figure S4. Spatial Distribution of the risk index (RI) in Suzhou soils by Kriging interpolation

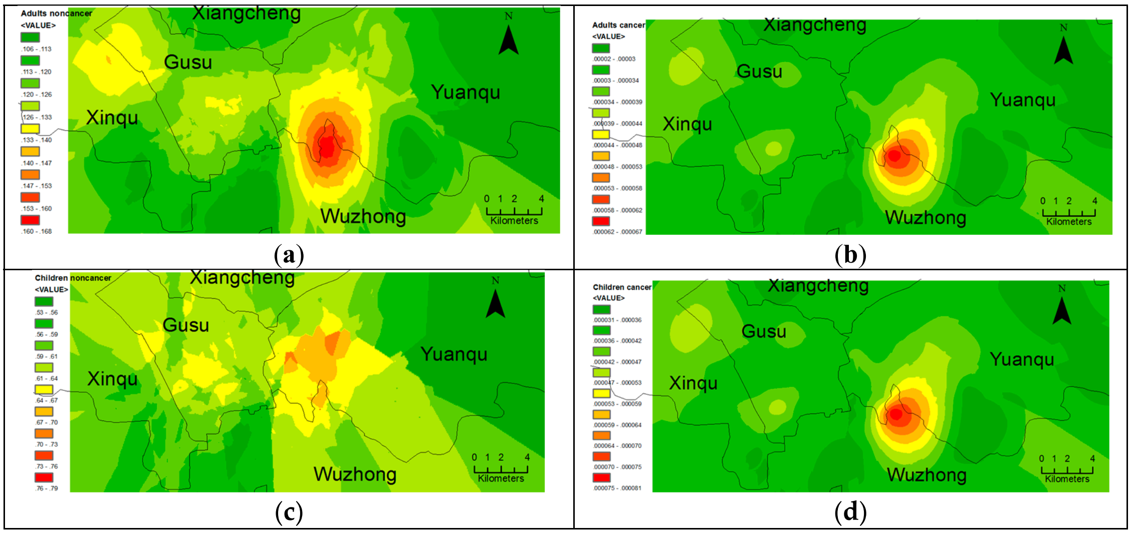

Figure S5. Spatial distribution of Hazard indices (HIs) and total carcinogenic risks (TCRs) for adults (**a** and **b**, respectively) and children (**c** and **d**, respectively) by Kriging interpolation.

1. Liu, Y.; Yue, L.L.; Li, J.C. Evaluation of heavy metal contamination and its potential ecological risk to the soil in taiyuan, china. (in Chinese) *J. Environ. Sci.* **2011**, *31*, 1285-1293.
2. Wang, M.; Markert, B.; Chen, W.; Peng, C.; Ouyang, Z. Identification of heavy metal pollutants using multivariate analysis and effects of land uses on their accumulation in urban soils in beijing, china. *Environ. Monit. Assess.* **2012**, *184*, 5889-5897.
3. Zhao, L.; Xu, Y.; Hou, H.; Shangguan, Y.; Li, F. Source identification and health risk assessment of metals in urban soils around the tanggu chemical industrial district, tianjin, china. *Sci. Total Environ.* **2014**, *s 468–469*, 654-662.
4. Yang, Z.; Lu, W.; Long, Y.; Bao, X.; Yang, Q. Assessment of heavy metals contamination in urban topsoil from changchun city, china. *J. Geochem. Explor.* **2011**, *108*, 27-38.
5. Liu, Y.L.; Zhang, L.J.; Han, X.F.; Zhuang, T.F.; Shi, Z.X.; Lu, X.Z. Spatial variability and evaluation of soil heavy metal contamination in the urban-transect of shanghai. (in Chinese) *Environ. Sci.* **2012**, *33*, 599-605.
6. Zeng-Fu, L.I.; Zhu, J.Y.; Wang, L.C. Heavy metal contents and their spatial distribution in urban soil of hefei city. (in Chinese) *Urban Environ. Urban Ecol.* **2009**.
7. Ying, L.; Shaogang, L.; Xiaoyang, C. Assessment of heavy metal pollution and human health risk in urban soils of a coal mining city in east china. *Hum. Ecol. Risk Assess.* **2016**, *22*, 1359-1374.
8. Cai, Q.Y.; Mo, C.H.; Li, H.Q.; Lü, H.; Zeng, Q.Y.; Li, Y.W.; Wu, X.L. Heavy metal contamination of urban soils and dusts in guangzhou, south china. *Environ. Monit. Assess.* **2013**, *185*, 1095-1106.
9. Lu, Y.; Gong, Z.; Zhang, G.; Burghardt, W. Concentrations and chemical speciations of cu, zn, pb and cr of urban soils in nanjing, china. *Geoderma* **2003**, *115*, 101-111.
10. Chao-Zhuang, X.I.; Dai, T.G.; Huang, D.Y. Investigation and assessment on pollution caused by soil heavy metals in changsha city,hunan province. (in Chinese) *Earth Environ.* **2008**.
